# Outcomes of a Medical Student Elective in Child and Adolescent Psychiatry: A Pilot Study

**DOI:** 10.1007/s40670-025-02381-0

**Published:** 2025-04-12

**Authors:** Larrilyn Grant, Lexi Singh, Mary A. Fristad, Jeff Barbee, Anna Kerlek

**Affiliations:** 1 Department of Psychiatry, Wright State University Boonshoft School of Medicine, Dayton, OH USA; 2 Division of Child & Family Psychiatry, Nationwide Children’s Hospital, Columbus, OH USA; 3 Department of Psychiatry and Behavioral Health, The Ohio State University College of Medicine, Columbus, OH USA

**Keywords:** Child and Adolescent Psychiatry, Medical students, Youth mental health, Suicide assessment, Biopsychosocial formulation, Social media usage

## Abstract

A majority of youth with mental health concerns lack access to psychiatric care, often relying on general practitioners who may feel unprepared. Enhancing medical education in child psychiatry is crucial. This study evaluated changes in medical students’ perspectives after participating in an interactive Child and Adolescent Psychiatry (CAP) elective. Significant improvements were found in students’ comfort assessing suicidal thoughts, evaluating mental health diagnoses, and understanding biopsychosocial factors. The findings suggest that participation in CAP electives may boost student confidence in handling youth mental health issues, highlighting the need for further studies on knowledge retention and career impact.

## Background

The number of youth with mental health (MH) concerns has been increasing [1]. Nearly 20% of youth in the United States (US) have a mental health condition [2]. Over 22% of US high school students have considered suicide, 10% have attempted, and 3% required medical treatment [3]. Addressing these issues effectively requires timely diagnosis and treatment, underscoring the importance of specialized care from child and adolescent psychiatrists (CAPs).

Despite this need, the severe shortage of US CAPs has been well-documented for over two decades [4]. Almost 75% of children live in counties without a CAP [5], and only 20% receive care from a MH practitioner [4, 5]. Given this shortage, primary care practitioners (PCPs) treat most youth with MH concerns. Recent studies show pediatric residents feel unprepared to treat pediatric depression and suicidality [6, 7]. Pediatric attending physicians report feeling limited comfort with providing psychoeducation and treating patients with MH conditions [8, 9], while PCPs cite insufficient training in MH diagnosis and management [10].

Medical school curricula provide minimal exposure to CAP with only 0–2% of training time dedicated to Child Psychiatry and only 85% of programs have a CAP faculty member [11, 12]. This is concerning as training enhances skills [13] and boosts CAP recruitment [14]. Nearly half of CAP fellows decide to subspecialize before or during medical school, highlighting this period as crucial for recruitment [15].

Given the CAP shortage and PCPs’ discomfort in managing pediatric MH, increasing CAP exposure in medical training is critical. This study evaluates medical students’ self-reported comfort and stigma regarding CAP after participating in an interactive virtual elective.

## Activity

A course focusing on CAP was created as a first- and second-year medical student elective at a Midwestern academic health center. This course occurred over 2 days with 8 h of direct teaching time. Due to the global pandemic, this course was administered via Zoom®. Topics included common MH diagnoses in youth, how disorders present uniquely compared to adults, a question-and-answer panel with CAPs, teenage use of digital media, patient conceptualization, discussion of CAP resources, and suicide assessments. Two mock interviews demonstrated how to assess suicide risk and explore gender dysphoria, with a CAP fellow role-playing a patient/family and providing real-time feedback on interview styles and questions.

Students completed a 10-item questionnaire created by the course directors to assess their comfort in CAP topics. Each item was scored using a 5-point Likert scale. Students received the questionnaire the weekend prior to the course. The questionnaire was repeated at the end of the elective. The pre- and post-tests for each item were compared using paired *t*-tests, with a Bonferroni correction to reduce the likelihood of type 1 error (alpha = 0.005). Student’ change scores were compared to determine if training level would impact results. Analyses utilized R Studio (R Core Team, 2021). The hospital’s Institutional Review Board declared this research exempt.

## Results and Discussion

**Fig. 1:**
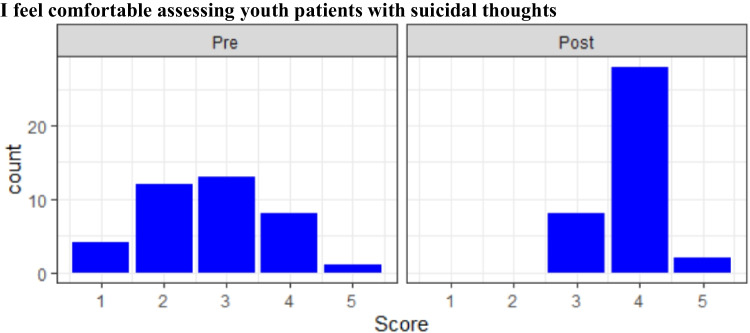
Results of pre (left) and post (right) course survey regarding comfort assessing youth with suicidal thoughts. *N* = 38

Twenty-three first-year and twenty-one second-year students completed the elective. Pre- and post-test surveys were completed by 38 of 44 students (86.4%: 19 first-year and 19 s-year students). Eight of 10 items showed significant improvement post-elective with large effect sizes (Table 1). Students reported more awareness of local youth MH resources, familiarity with a biopsychosocial conceptual model, how to evaluate youth with MH concerns, how to identify common MH diagnoses in youth, how to assess youth with suicidal ideation, what CAP careers entail and their training pathway, and youth use of digital media. A minority of students endorsed feeling “confident” or “very confident” about assessing suicidal ideation pre-course, whereas a majority did so post-course (Fig. 1). Twelve students reported feeling “very confident” post-course versus none pre-course in identifying MH diagnoses in youth.

**Table 1:** Paired results from pre- and post-course survey from first- and second-year medical students. *N* = 38/44

Question	*Pre-test* [95% confidence interval]	*Post-test* [95% confidence interval]	*Score Diff*	*p*	*Cohen’s d* [95% confidence interval]
I am aware of behavioral health resources available at Nationwide Children’s Hospital	2.29 [2.03, 2.55]	4.37 [4.21, 4.53]	2.08	< *.005*	3.59 [2.87, 4.32]
I am aware of the biological, social, developmental, and psychological impacts on patient care	3.76 [3.52, 4.01]	4.55 [4.39, 4.72]	0.79	< *.005*	1.59 [1.07, 2.10]
I am familiar with how to evaluate child and adolescent patients with potential mental health diagnoses	2.53 [2.26, 2.79]	4.13 [3.98, 4.29]	1.60	< *.005*	2.56 [1.95, 3.17]
I am interested in becoming a child and adolescent psychiatrist	3.32 [3.10, 3.53]	3.61 [3.36, 3.85]	0.29	*n.s*	0.59 [.13, 1.05]
I can identify common mental health diagnoses in children and adolescents	3.26 [2.95, 3.58]	4.21 [4.01, 4.41]	0.95	< *.005*	1.29 [.8, 1.78]
I feel comfortable assessing youth patients with suicidal thoughts	2.74 [2.41, 3.07]	3.84 [3.68, 4.00]	1.10	< *.005*	1.64 [1.12, 2.16]
I have a general understanding of what a child and adolescent psychiatrist can do in their professional career	3.13 [2.85, 3.41]	4.47 [4.31, 4.64]	1.34	< *.005*	2.09 [1.53, 2.65]
I know how most contemporary teens use digital media	4.05 [3.84, 4.27]	4.53 [4.36, 4.69]	0.48	< *.005*	1.11 [0.63, 1.59]
I understand the various pathways that exist in order to become a child and adolescent psychiatrist	2.42 [2.16, 2.68]	4.32 [4.10, 4.53]	1.90	< *.005*	2.81 [2.18, 3.45]
Patients with psychiatric concerns are more difficult to assess and treat compared to other patients	3.18 [2.92, 3.45]	3.21 [2.91, 3.51]	0.03	*n.s*	0.04 [0,.49]

Note: Interpretation of Cohen’s *d* is as follows: *d* = 0.20, small effect size, 15% nonoverlap of distributions; *d* = 50, medium effect size, 33% nonoverlap of distributions; *d* = 0.80, large effect size, 50% nonoverlap of distributions. Most items had a very large effect size

Two items showed no significant change. While interest in becoming a CAP increased slightly (*d* = 0.59, *n.s.*), perceptions of the difficulty in treating youth with mental illness remained essentially unchanged.

No significant differences were found between first and second-year students in baseline knowledge or confidence. Future studies should assess the optimal timing for this educational intervention.

This pilot study shows that a virtual CAP elective improves confidence in evaluating CAP disorders and suicidal ideation. Given suicide’s prevalence, teaching future physicians to assess suicidal patients is crucial. Students responded positively to didactics followed by simulated patient interviews, a method previously shown to enhance clinical skills [16–18]. Future studies should assess not only confidence but also knowledge retention and decision making.

Our findings align with prior studies showing brief interventions improve confidence [16], with longer postgraduate courses enhancing comfort [13]. Given the reliance on non-CAP practitioners for pediatric MH care, future studies should explore the most effective ways for increase PCP confidence.

Utilizing a biopsychosocial model improves health outcomes, yet many trainees struggle with its application [19]. Protocols are being developed to teach the biopsychosocial model to trainees evaluating certain medical conditions [20]. Future studies should continue to evaluate how education and application of patient conceptualization improves patient outcomes.

A newer concern in CAP is the increasing use of social media in children and adolescents [21]. Children and adolescents who spend more than 3 h per day on social media may be at an increased risk of MH problems [22], and a recent survey showed that teenagers spend an average of 4.8 h per day on social media [23]. Despite the perceived importance of counseling youth on social media usage, many practitioners do not feel comfortable in this area [24]. However, PCPs who have social media counseling training are more likely to counsel youth on social media use and subsequently improve youth online safety behaviors [25]. Our study revealed students had improved self-assessed understanding of how youth utilize social media. Future studies should evaluate how to improve medical students’ ability to counsel about usage concerns in youth on social media.

Given the burden of youth MH treatment on non-CAP practitioners, it is important that students and physicians are aware of resources to assist them in treating these patients. Our study showed improvement in awareness of local MH resources. National resources previously have been generated by the Association of Directors of Medical Student Education in Psychiatry [26] and the American Academy of Child and Adolescent Psychiatry (https://www.aacap.org/) and can be shared with students.

While interest in CAP did not increase significantly, a small rise in interest suggests that more exposure may enhance recruitment. While our study and others have shown improved knowledge of how to become a CAP physician, few methods have resulted in improved interest in entering the field [26–28]. Surveys of early career CAP attendings and CAP fellows suggest recruitment rates may increase with early career child psychiatry exposure, improved compensation, and incentives to address physician debt [29]. Future studies should continue to address and evaluate pre-existing barriers to CAP recruitment in medical school.

Confidence in assessing youth mental health disorders improved, though this did not reduce students’ perceived difficulty in treating them. Previous studies indicate that pre-clinical education can reduce mental health bias [30], warranting further exploration of medical students’ perceptions of youth mental health as a barrier to entering CAP.

Limitations of this study include a small sample size and lack of demographic information, making results difficult to generalize to other medical student populations. Since this course is voluntarily selected by students, students in the study may already have a general interest in MH, which could have impacted results. The 10-item survey was developed to examine each item separately rather than as a combined construct. Because of this, the instrument is not appropriate for traditional validation and reliability analyses. Additionally, this study evaluated self-reported change in confidence, which may not correspond with actual improvement in knowledge, effectiveness, or skill retention. Lastly, our study lacked long-term follow-up with students; therefore, it is unknown if students’ increased confidence persisted beyond the immediate post-course period or if the course influenced students’ long-term career outcomes.

In conclusion, this study demonstrated the feasibility and preliminary effectiveness of a virtual CAP elective for medical students. Participants reported increased confidence in evaluating and identifying CAP disorders, as well as in assessing youth with suicidal ideation. Incorporating high-yield topics and mock interviews into CAP curriculum development could further enhance students’ confidence in managing CAP-related disorders. This curriculum can be introduced early in medical school increasing exposure to an often-unknown specialty. Given the critical shortage of CAP physicians, equipping future physicians across all specialties with skills to evaluate and treat pediatric patients with mental health concerns is essential. Future research should focus on assessing knowledge acquisition, retention, long-term career trajectories, and sustained confidence in managing youth with mental illness and suicidality among participants in CAP electives.
